# Recalcitrant Dry Eye Disease in a 31-Year-Old Female: Favorable Outcomes Following Complete Ocular Lavage Facilitated by an Irrigating Eyelid Retractor

**DOI:** 10.7759/cureus.78554

**Published:** 2025-02-05

**Authors:** Sathi Maiti, Srinivas Sai A Kondapalli, Laura M Periman

**Affiliations:** 1 Ophthalmology, Periman Eye Institute, Seattle, USA; 2 Retina, Everett and Hurite Ophthalmic Association, Pittsburgh, USA

**Keywords:** dry eye disease, meibomian gland dysfunction, novel therapies, ocular surface disease, ocular surface inflammation

## Abstract

This report explores the management of an otherwise healthy 31-year-old Caucasian female with chronic, refractory dry eye disease (DED) unresponsive to extensive conventional therapies. The initial treatment included artificial tears, cyclosporine, hypochlorous acid spray, and thermal eyelid pulsation, which provided limited relief. Progressive therapeutic interventions, such as intense pulsed light, varenicline nasal spray, perfluorohexyloctane drops, platelet-rich plasma, and topical antibiotics, resulted in only modest improvement over 15 months. Persistent symptoms and corneal staining prompted the implementation of a novel treatment: high-pressure ocular surface lavage using an irrigating eyelid retractor (Rinsada®) with preservative-free saline. Post-lavage, the patient experienced immediate and significant symptomatic relief, with marked improvement in corneal staining noted on slit lamp examination. Continued improvement was observed over two weeks following the procedure.

This report underscores the potential of high-pressure saline irrigation for addressing inflammatory mediators and biofilm on the ocular surface. The irrigating eyelid retractor enabled precise and effective lavage of the palpebral conjunctiva and fornices, reducing the inflammatory load and resetting the ocular surface. This technique represents a promising adjunctive therapy for recalcitrant DED, offering rapid symptom relief and improved clinical outcomes. Further studies are warranted to validate these findings and optimize patient care.

## Introduction

Dry eye disease (DED) is a multifactorial disease of the ocular surface characterized by a loss of tear film stability resulting in ocular discomfort, tearing, and fluctuations in visual quality. The incidence of the disease has been reported to be as high as 50% of the population [1], with significant implications for patients’ vision and overall quality of life. While the management of DED has significantly advanced, treatment options often fail to provide adequate disease control. 

The treatment for DED is variable and depends on the underlying mechanism of the disease. DED can be grossly categorized into two groups: evaporative dry eye and aqueous-deficiency dry eye [2]. While aqueous deficiency DED is caused by a poor output mechanism from the lacrimal glands, evaporative dry eye is thought to be secondary to a failure of tear film stability and elevated ocular surface inflammation [3]. In clinical practice, it is uncommon to find a patient with solely one of these processes being causative for their symptoms, and both states of disequilibrium manifest simultaneously instead. There are various methods to diagnose and assess DED, the cornerstone of which is a clinical examination. A thorough examination can be augmented with additional diagnostic testing, including inflammatory point-of-care testing of matrix metalloproteinase (MMP)-9, osmolarity testing, and tear film analysis [2,3].

The treatment of DED is also based on the severity of the disease and underlying pathophysiology. Preliminary steps in the management of disease may involve patient education and environmental modifications including reducing screen time and the use of an ambient humidifier. Identifying and removing any iatrogenic causes of the DED including systemic or topical medications and/or supplements can be coupled with the use of topical ocular lubricant. Ocular lubricants are thought to work by reducing ocular surface inflammation and osmolarity by dilution [4]. Lid margin hygiene, which includes warm compresses and lid scrubs with or without hypochlorous acid, can be useful for the treatment of meibomian gland dysfunction and Demodex-associated blepharitis [5]. Lotilaner ophthalmic solution 0.25% was recently introduced into the US market for the treatment of Demodex blepharitis [6]. These treatment modalities attempt to reduce biofilm burden at the lashes and lid margin to stabilize the lipid layer of the tear film, reducing the evaporative component of DED.

There are a variety of ways to treat ocular surface inflammation, which include topical corticosteroids, cyclosporine, lifitegrast, doxycycline, and autologous serum. The mechanisms of action of these modalities vary from the reduction in T lymphocyte recruitment and MMP 9 expression to the improvement in tear film stability [7]. Antibiotic drops and ointment reduce the microbial load of the tear film, which can result in a more stable ocular surface. In-office procedures including intense pulse light therapy and heating and expression of the meibomian glands have also been effective for some patients in the management of, demonstrating improvements in both examination findings and symptoms [8].

However, even with all modern therapies, patients may still suffer from refractory DED. This report discusses the clinical course of a female patient with refractory DED, who, despite undergoing multiple therapeutic interventions, continued to experience persistent signs and symptoms of the disease. She ultimately underwent a novel treatment: an ocular lavage with an irrigating eyelid retractor (Rinsada®). The retractor aims high-pressure irrigation fluid to the palpebral conjunctiva, bulbar conjunctiva, and conjunctival fornix simultaneously. Based on our findings, complete ocular lavage with an irrigating eyelid retractor should be considered in the management of DED. 

The patient gave informed consent for the publication of this case report and associated images, with the understanding that all identifying information would be removed to maintain confidentiality.

## Case presentation

A 31-year-old Caucasian female presented in February 2023 with a long-standing history of DED symptoms including burning, tearing, and foreign body sensation in both eyes. Her treatment at the presentation included over-the-counter artificial tears QID OU, ointment at bedtime, cyclosporine ophthalmic solution 0.05% BID OU, and hypochlorous acid 0.01% spray QD. She had also undergone one thermal pulsation of the eyelids by an outside physician with minimal improvement in her signs or symptoms. She was on no systemic medications and was otherwise healthy. 

**Figure 1:**
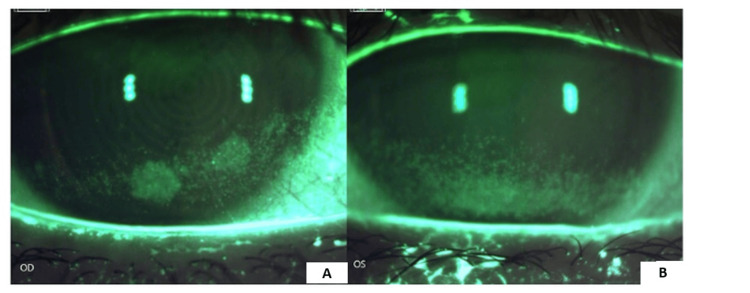
Corneal staining on presentation First visit - February 2023. The patient’s right eye (A) and left eye (B) illustrate significant superficial punctate keratitis of the lower one-third of the cornea and limbus

On external examination, there was mild lagophthalmos OU with thick, turbid, and poorly expressible meibomian glands. On slit lamp examination, the patient had dense inferior superior punctate keratitis OS>OD (Figures 1A, 1B) and a tear breakup time (TBUT) of four seconds OU. Corneal esthesiometry was 4 cm with Cochet-Bonnet. The rest of her ophthalmic examination was unremarkable. The patient was diagnosed with meibomian gland dysfunction and dry eye syndrome OU.

The patient was started on omega-3 supplementation, varenicline nasal solution BID, a series of four intense pulse light treatment sessions, cyclosporine 0.05% QID OU, hypochlorous acid spray QD, preservative-free ointment QHS, and a sleep mask to help with nocturnal lagophthalmos. In addition, she was instructed to start a two-week course of moxifloxacin gtt OU BID and mupirocin BID in each nasal vestibule.

Upon return four months later in June 2023, the patient demonstrated improvement in her dense superficial punctate keratitis (SPK), which was now noted to be trace OU. She was advised to continue varenicline nasal solution BID, omega-3 supplementation, and intense pulsed light as needed. Eight months later (February 2024), the patient returned with worsening symptoms. Exam illustrated dense SPK now OD > OS. She was prescribed perfluorohexyloctane ophthalmic solution BID, cyclosporine ophthalmic solution 0.1% BID, lotilaner ophthalmic solution BID, and another course of topical antibiotics: moxifloxacin TID x seven days, mupirocin ointment to nasal vestibules BID x two weeks. 

**Figure 2:**
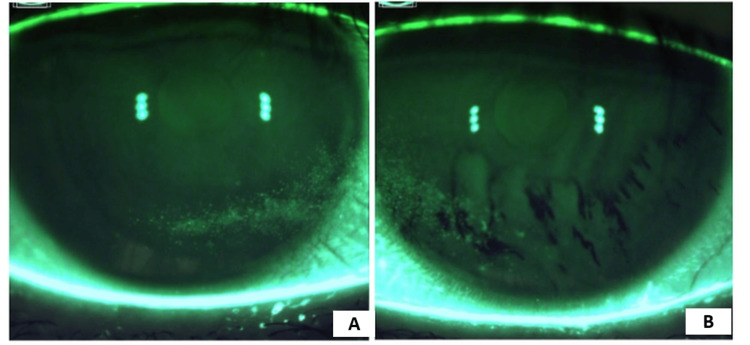
Corneal staining with active pharmaceutical intervention A: The image shows the right eye of the patient with persistent inferonasal superficial punctate keratitis on active pharmaceutical management. 2B: The image illustrates the left eye of the patient with persistent inferonasal superficial punctate keratitis and irregular staining pattern on active pharmaceutical management

The patient returned in May 2024 with minimal improvement in her symptoms and signs of DED. At this point, she was prescribed autologous platelet-rich plasma QID x three months. She again returned for examination in June 2024. At this stage, she had finished a second course of lotaliner ophthalmic solution, perfluorohexyloctane ophthalmic solution, preservative-free artificial tears, varenicline nasal solution, and platelet-rich plasma drops with ~20% improvement in dense SPK (Figures 2A, 2B).

Given persistent signs and symptoms of ocular surface disease, the decision was made to perform high-pressure irrigation lavage of the entire ocular surface using an irrigating eyelid retractor that day; 15 cc 0.9% preservative-free, sterile normal saline solution was used with the irrigating eyelid retractor. 10 cc of saline was used in the superior palpebral conjunctiva and fornix and 5 cc of saline was used in the inferior palpebral conjunctiva and fornix of each eye. 

**Figure 3:**
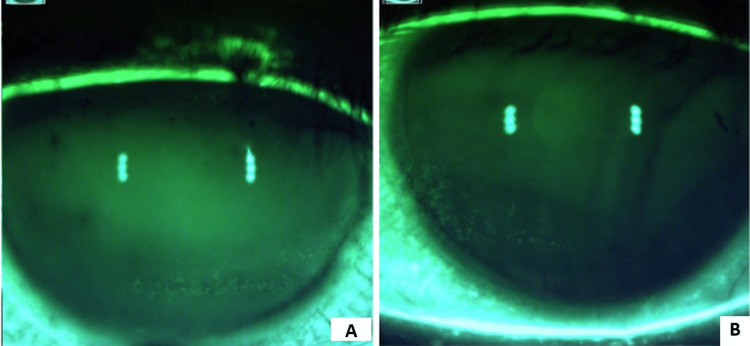
Corneal staining one day post complete ocular lavage The right eye (A) and left eye (B) of the patient with reduced superficial keratitis and improved staining pattern one day after complete ocular lavage with irrigating eyelid retractor

**Figure 4:**
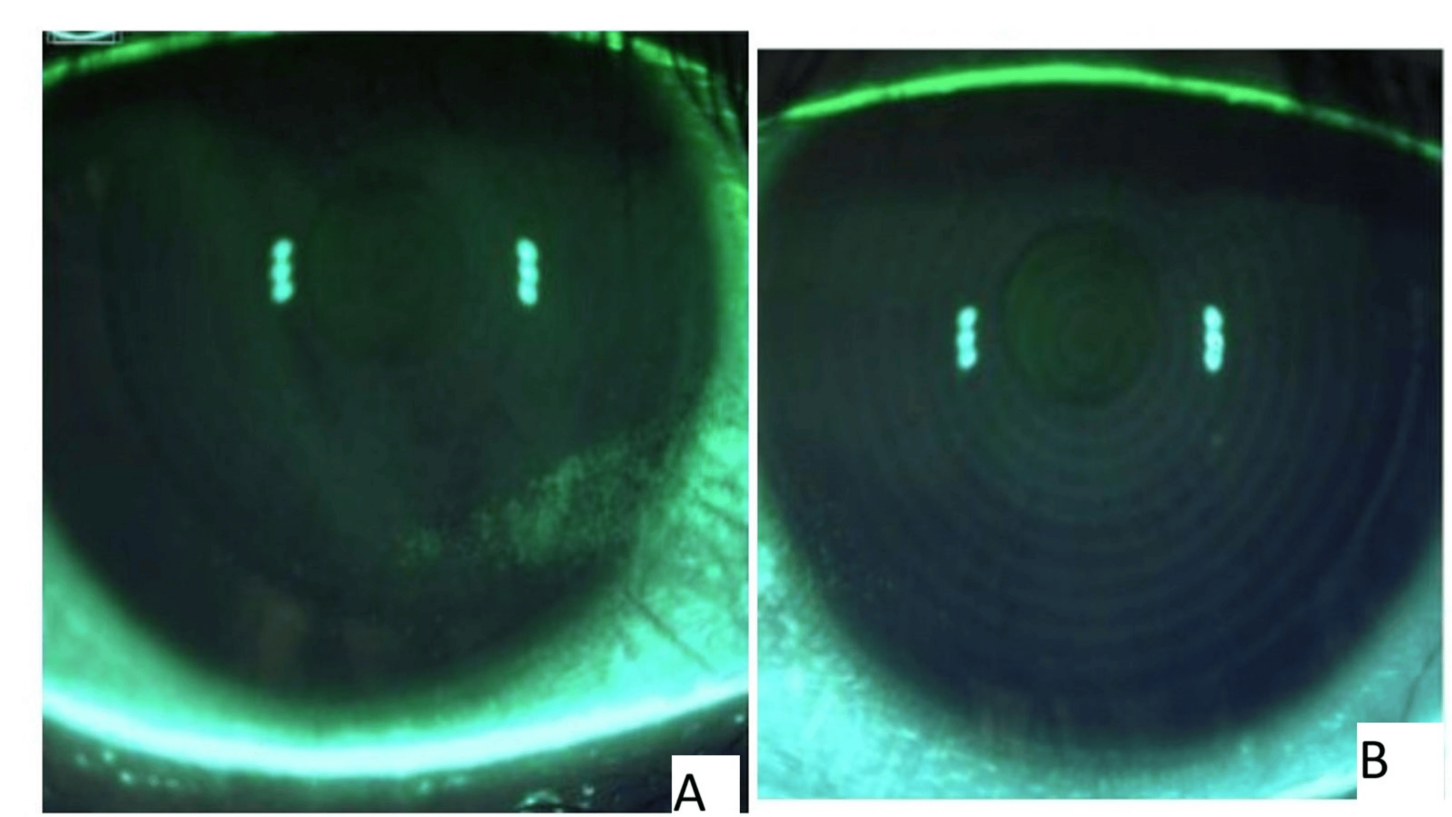
Corneal staining approxiamately two weeks post complete ocular lavage The right eye (A) and the left eye (B) of the patient with nearly resolved superficial keratitis and improved staining pattern 13 days after complete ocular lavage with the irrigating eyelid retractor

The patient returned to the clinic the next day with significant subjective improvement in her symptoms. Moreover, on repeat staining on slit lamp examination, there was reduced staining and SPK noted on the cornea (Figures 3A, 3B). At the patient’s most recent visit, 13 days following ocular lavage with the irrigating eyelid retractor, there was continued improvement of corneal staining (Figures 4A, 4B). The patient was maintained on cyclosporine BID, varenicline nasal spray BID, platelet-rich plasma drops BID, perfluorohexyloctane QID, and preservative-free artificial tears. She was found to be asymptomatic. 

## Discussion

DED is a multifactorial inflammatory condition of the ocular surface characterized by irritation, redness, pain, photophobia, and/or fluctuations in vision [1]. Interventions in DED target various pathophysiologies that trigger symptomatic disease. In our patient with chronic, recalcitrant DED, the therapies spanned the gauntlet. She initially presented while on anti-inflammatory therapy, cyclosporine ophthalmic solution twice a day, as well as topical hypochlorous acid for the eyelashes, which can remove or reduce biofilm burden on the lash margin. The patient's course continued with a variety of different topical pharmaceuticals with varied mechanisms of action, e.g., to reduce evaporation, to destroy mite infestation of the lashes, or to reduce T-cell mediated inflammation. These treatments failed to improve the patient's symptoms or corneal findings satisfactorily.

**Figure 5:**
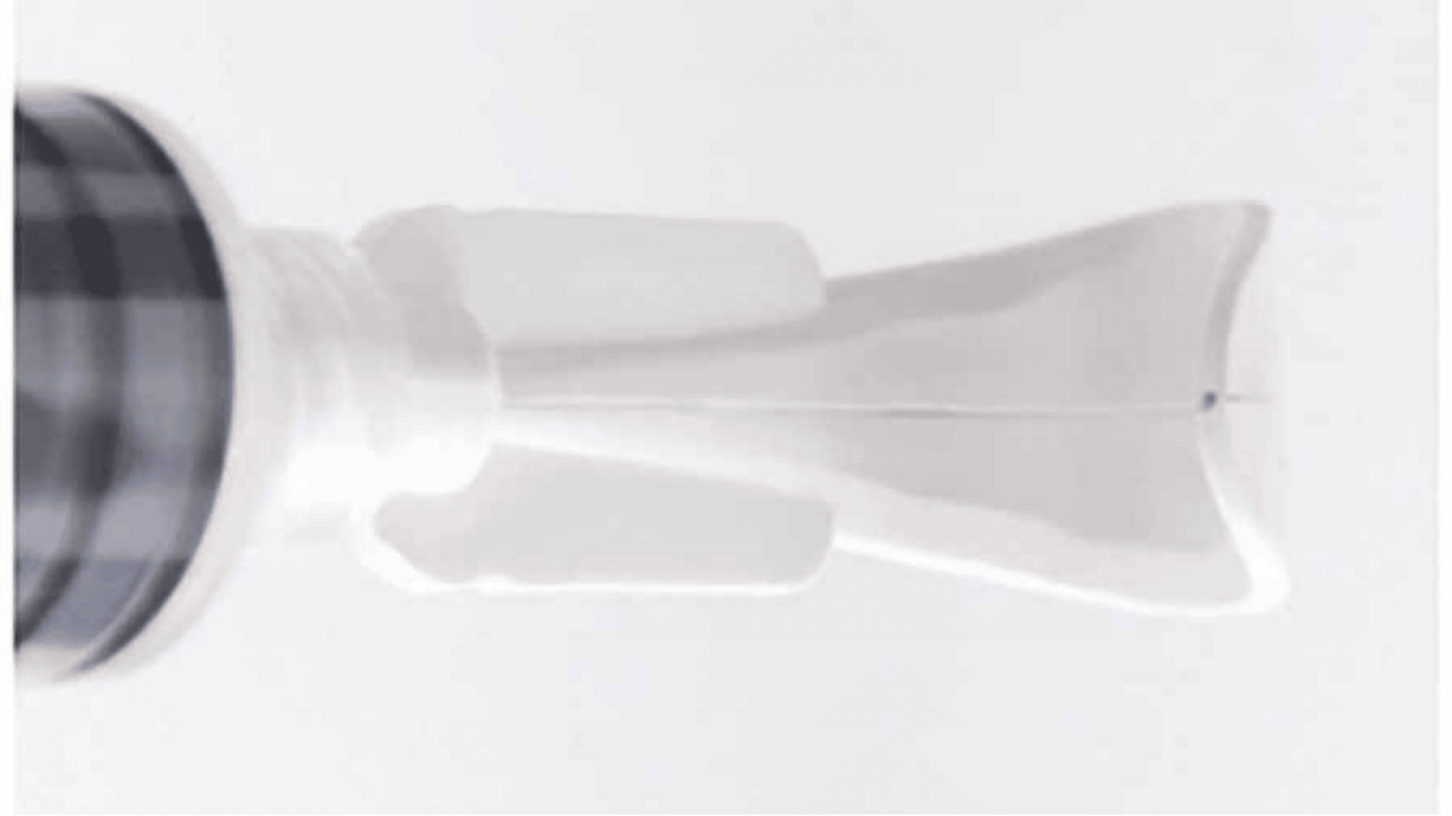
Irrigating eyelid retractor View of the irrigating eyelid retractor with the distal port that aims fluid at the fornix

The use of the irrigating lid retractor offers a novel avenue to treat patients with ocular surface disease. The device is a sterile, single-use medical device with ports on the distal end that aim high-pressure fluid at the bulbar conjunctiva, palpebral conjunctiva, and conjunctival fornix simultaneously (Figure 5).

The hypothesis in this treatment is that the tear film is infiltrated with a high degree of foreign or pathologic mediators. These include cytokines, MMP, microparticulate matter, and biofilm endotoxins. These pathologic mediators harbor or are trapped within the palpebral conjunctiva and fornix [10]. Dilution and removal of the tear film inflammatory mediators are thought to partially be the mechanism of action of artificial tears; however, the artificial tear’s volume and therapeutic location are insufficient in some patients with high inflammatory load in their tear film. As such, by using high pressure and volume irrigation specifically targeting the palpebral conjunctiva and fornix, we were able to reduce this inflammatory load that sits in the tear film.

A randomized controlled clinical trial has shown that complete ocular surface lavage with the irrigating lid retractor can reduce MMP-9 by 72% and, in some patients, keep them at negative MMP-9 in their tear film for three months [11]. The device-facilitated lavage was statistically superior to standard lavage. Moreover, other studies assessing the combination of microblepharoexfoliation and complete ocular lavage with the irrigating eyelid retractor showed improvement in patient DED symptoms compared to microblepharoexfoliation alone [12]. The clinical application of the ocular lavage with an irrigating eyelid retractor is often repeated on an as-needed basis depending on patient symptoms. Alternatively, physicians can also repeat lavage on a bi-annual basis. In the United States, the procedure is not billed to insurance but an out-of-pocket expense. Over 4800 devices have been within the United States at the date of publication without any safety concerns [13]. This idea is not novel in health care; high-pressure irrigation is used elsewhere in the body to facilitate greater homeostasis. For example, high-pressure irrigation of the nasal sinuses is an effective mechanism to reduce allergen load in patients suffering from allergic rhinitis [14]. 

Future research should focus on expanding the evidence base by conducting larger, multi-center case series. This would provide more robust data regarding the treatment's efficacy, potential side effects, and data generalizability across diverse patient populations. By increasing the sample size and incorporating diverse patient demographics, future studies can help clarify the broader applicability of these findings and provide more definitive guidance for clinical practice.

## Conclusions

We described a recalcitrant case of DED that was successfully managed with a complete ocular lavage with an irrigating eyelid retractor. The ocular lavage provided a different mechanistic option to improve the tear film, i.e., reducing tear film inflammation, which led to an improvement in the patient’s corneal staining and symptomatology.
